# What's keeping kids up at night? How psychosocial stressors exacerbate the relationship between sleep and mental health

**DOI:** 10.1002/puh2.95

**Published:** 2023-06-21

**Authors:** Nipher Malika, Tori R. Van Dyk, Qais Alemi, Juan Carlos Belliard, Catherine Fisher, Larry Ortiz, Susanne Montgomery

**Affiliations:** ^1^ RAND Corporation Santa Monica California USA; ^2^ School of Behavioral Health Loma Linda University Loma Linda California USA; ^3^ School of Public Health Loma Linda University Loma Linda California USA

**Keywords:** adolescents, bullying, children, discrimination, mental health, safety, sleep

## Abstract

**Background:**

Although it is well established that healthy sleep promotes positive mental health, little is known about how sleep operates in children and adolescents who experience a range of psychosocial stressors. This study examined the association between sleep duration and serious mental illness (SMI) and how this pathway is moderated by psychosocial stressors (discrimination, bullying, and perceived school and neighborhood safety).

**Methods:**

A cross‐sectional study was conducted among students in a California school district serving a low‐income community in 2019–2020. A non‐probability convenience sampling method was used, and surveys were administered in English, in a single class period. Basic descriptive statistics and a hierarchical linear regression analysis were used.

**Results:**

Students (*n* = 24,439) in grades 5–12 were surveyed. An average of 18.7% of the students reported having SMI; however, distribution increased by grade from 13.6% in 5th grade to 24.5% in 11th grade. Sleep duration was inversely associated with SMI, as the hours of sleep decreased, the risk of SMI increased. The negative effect of poor sleep on SMI was further exacerbated by perceived discrimination at school (*β* = 0.13, *p* < 0.001), feeling unsafe in one's neighborhood (*β* = 0.32, *p* < 0.001), feeling unsafe at school (*β* = 0.23, *p* < 0.001), and being bullied at school (*β* = 0.54, *p* < 0.001).

**Conclusion:**

This study was demonstrated that increased sleep among children and adolescents was associated with reduced SMI. However, in the presence of psychosocial stressors (discrimination, bullying, and perceived school and neighborhood safety), the effect of sleep on SMI was moderated and despite increased sleep.

## Graphical abstract

**Graphical Abstract**: Increased sleep among children and adolescents was associated with reduced serious mental illness (SMI). However, in the presence of psychosocial stressors (discrimination, bullying, and perceived school and neighborhood safety), the effect of sleep on SMI was moderated and despite increased sleep, experiencing psychosocial stressors was found to increase one's SMI.

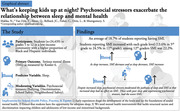

## INTRODUCTION

Mental illness, along with stress and distress, among children and adolescents is a major public health concern [1, 2, 3]. Today, a total of 13%–20% of children living in the United States experience a mental disorder in a given year [4], and it is estimated that by the time of adolescence, nearly half of youth will have experienced at least one mental health condition, with over 20% experiencing severe impairment [5]. This rate is on the rise as research indicates that unique factors, such as the increasing frequency of mass shootings, high use of social media, and the challenges brought on by COVID‐19, exert a psychological toll on children and adolescents [6, 7, 8].

Mental health disorders among children and adolescents have become common with 73.8% presenting with both depression and anxiety [1]. Mental health disorder significantly affects their maturation by interfering with cognitive, academic, social, and emotional development [9, 10]. According to the American Psychiatric Association, among minoritized populations, mental disorders are reportedly similar and in some cases, lower in comparison to Whites [11]. However, several studies have shown evidence of misdiagnosis of mental health disorders among racial/ethnic minority youth [12, 13, 14, 15]. In addition, minoritized individuals also have delayed diagnosis, less access to treatment, and more likely to receive poorer quality of care for their mental health problems [15, 16]. As a result, consequences of mental health disorders in non‐Whites lasts longer when compared to Whites [1, 11]. Furthermore, in addition to the stressors all children and adolescents face that contribute to their mental health disorders, children and adolescents of minoritized and low‐income status have additional stressors contributing to higher anxiety such as discrimination, poor safety (at school and in their neighborhoods), and bullying [17, 18, 19, 20].

The COVID‐19 pandemic pointed anew to glaring gaps within schools’ ability to equitably meet the needs of all students especially as it relates to mental health [21]. School districts all over the country are trying to address these inequities and understand how additional psychosocial stressors (e.g., discrimination, safety, bullying) contribute to mental health in order to intervene and enhance student mental health and academic performance [22, 23, 24]. Efforts to address mental health have involved addressing sleep deficits among children and adolescents [25, 26]. Sleep is a physiological state regulated by a specific pattern of cerebral electric activity between sleep and wakefulness or circadian rhythm [27]. Disturbances and chronic disruption in the sleep–wake cycles are associated to various metabolic, psychiatric, and neurodegenerative disorders [28]. Among children and adolescents, sleep disturbances are associated with many mental health and behavioral disorders [29, 30]. However, studies have also found that adequate sleep and proper regulation of the sleep–wake pattern positively play a restorative role on the immune system and brain function [31, 32].

With most United States schools on average having a starting time of 8 a.m. (with some schools beginning earlier than 7:30 a.m. and others after 9 a.m.), nearly 60% of middle schoolers and 70% of high schoolers were reportedly not getting enough sleep [33, 34, 35]. Several studies have shown that current and early school start times are associated with students getting less sleep due to the biology of sleep cycles of children and adolescents [36, 37, 38]. For instance, at the beginning of puberty, most adolescents experience later sleep onset and wake times, and as a result, the average teenager does not fall asleep until 11 p.m. and thus would do best by waking up at 8 a.m. [36, 38]. Therefore, with states like California changing school start times and many other states considering a change in start time [39, 40], it is important for schools to see the role of sleep in mental health, and even more so, its interplay with psychosocial stressors such as perceived racial/ethnic discrimination at school, bullying at school, and poor safety at school and in the neighborhoods student live in.

Thus, the aim of this study was to assess the role sleep, discrimination, safety, and bullying have on serious mental illness (SMI) in a child and adolescent sample of students. In this manuscript, we model sleep as a predictor of SMI while examining the potential deleterious effects of psychosocial stressors (i.e., perceived racial/ethnic discrimination at school, bullying at school, and feeling unsafe at school and at home) on this relationship in an under‐resourced, majority minority region of southern California. Our objectives were to: (1) assess sleep adequacy/deficit of racially/ethnically minoritized students, (2) understand the distribution of psychosocial stressors, and (3) examine how psychosocial stressors moderate the relationship between sleep and mental health.

## METHODS

### Study design, subjects, and sampling

A cross‐sectional study was conducted in a school district serving 54,841 students to assess the role sleep, discrimination, safety, and bullying have on SMI. The inclusion criteria were students attending a specified school district, students in grades 5–12, and students who provided both parental consent and student assent.

Of those eligible students between grades 5 and 12 (28,588), a sample size was determined by using the actual student enrollment numbers in the previous year for each grade level, Cochran's formula and assuming a 95% confidence interval and 5% margin of error for each grade level. This gave us a minimum sample size of 2779 total students between grades 5 and 12 and an average of 345 students per grade.

This study used a non‐probability convenience sampling method whereby all eligible students were sent a description of the study, the data collection processes, a parental consent, and student assent form. Those who returned the consent and assent forms participated in the study. Students and parents were also informed that participation in the survey was voluntary and that the student responses would remain anonymous. This study was approved by the Loma Linda University Institutional Review Board, and all participants provided written informed assent and informed consent from parents.

### Setting

Participants were students selected from a school district serving a low‐income community in Southern California. Compared to the demographics of many Californian public‐school students, this school district is more racially diverse with a higher proportion of Black and Hispanic students. Moreover, approximately 90% of the students are eligible for free and reduced lunch reflecting the socioeconomic disadvantage of this community. The school district serves 54,841 students from kindergarten to grade 12 and has 21% of English language learners of whom 20% identify Spanish as their first language.

This school district is in a county with approximately 2.1 million people where 55% of its population identify as Hispanic or Latino. Approximately 20% are foreign born persons, 42% have a language other than English spoken at home, and the child poverty rate of this county is at 18% which is higher than the national poverty rate of 16.9%.

Encompassed in the district's goal to create a culture of health, the district partnered with a local university and an external consulting company to collect yearly surveys to assess the health, wellness, and safety of the students. The data used in this study is from the 2019–2020 school year and was collected in the fall of 2019.

### Data collection

Each school in the district worked out its own school schedule in a given month in the fall of 2019, and its students were then assigned to a class in which they would take the survey on computers. Surveys were administered in English under each student's school identification number, in a single class period, and administered by a third‐party vendor. In an effort to minimize impact on instructional time, the numbers of questions asked were limited so that the survey could be taken by all students in 1 h. A teacher, teacher's assistant, substitute teacher, or school administrator made themselves available during survey administration, to answer questions students may have while taking the survey. Due to the fact that the surveys were conducted under each student's identification number, it made it easier for survey responses to be linked to the student's record (e.g., attendance rate, suspension, and grades). However, a de‐identified dataset was used for analysis.

### Instrument

The survey instrument measured school climate, social emotional learning, health and risky behaviors of the students and the adults at home, home and neighborhood environment, physical and mental health, food and nutrition, and discrimination.

#### Serious mental illness (SMI)

A globally used measure in epidemiological studies is the Kessler6 (K6), a nonspecific distress scale that screens for possible SMI [41, 42]. The scale was designed to identify those within a clinically significant range indicative of nonspecific distress in an effort to maximize the ability to discriminate cases of SMI from non‐cases [42]. Items on the K6 are based on a 30‐day reference period generating a scale range of 0–24 with higher scores indicative of higher occurrence of SMI and “Persons with a score of 13 or greater are likely to be experiencing SMI” [42].

#### Sleep duration

Sleep duration was assessed using a single item question. As informed by the *Youth Risk Behavioral Survey Questionnaire* [43, 44], we asked participants “On an average school night, how many hours of sleep do you get?” Responses included, “4 or less hours,” “5 h,” “6 h,” “7 h,” “8 h,” “9 h,” and “10 or more hours.”

#### Perceived racial/ethnic discrimination, school and neighborhood safety, and bullying

We assessed perceived racial/ethnic discrimination at school using one item from the California Healthy Kids Survey [45]. Participants were presented the statement “I have been disrespected or mistreated by an adult at this school because of my race, ethnicity or nationality.”

Bullying at school was assessed with six questions: “during the past 12 months, how many times on school property have you … (a) been pushed, shoved, slapped, hit, or kicked by someone who wasn't just kidding around? (b) been afraid of being beaten up? (c) had mean rumors or lies spread about you? (d) had sexual jokes, comments or gestures made to you? (e) been made fun of because of your looks or the way you talk? (f) had your property stolen, or deliberately damaged, such as your car, clothing or books?”

Safety in school was assessed with the following question: “How safe do you feel when you are at school?” The *Safety in your neighborhood* item was adapted from the California Healthy Kids survey, which asked participants, “how safe do you feel in the neighborhood where you live?” Both safety items used likert scales ranging from 1 = “very unsafe” to 5 = “very safe.”

#### Sociodemographic controls

We considered a number of sociodemographic characteristics, including gender, race (Black/African American, Hispanic, White, other races), grade in school (5th–12th), sleeping with electronics, and income. As a proxy for income, we assessed qualification for free lunch. Those categorized into “other races” included students identifying as Asian, American Indian or Alaska Native, Native Hawaiian or Pacific Islander, Filipino, two or more races, and those who did not report a race. Sleeping with electronics was important as a covariate to control for and the question asked, “how often do you sleep with one of these devices (an Xbox, PlayStation, an iPad or other tablet, a smartphone) near where you sleep, such as in your bed or next to your bed?” The choices ranged from 0 to 7 days a week and were dichotomized as “no” not sleeping with electronics (0 day; coded as 0)) and “yes” sleeping with electronics (1–7 days a week; coded as 1). Sleeping with electronics has been found to be associated with reduced sleep duration and quality [46] and therefore as a confounder, including it in the regression, allows the model to separate its effects from those of the explanatory variables of interest.

### Data analysis

SAS, version 9.4, was used for all analyses. First, for descriptive purposes, we ran generated means and standard deviations for continuous variables, and frequencies and percentages for categorical variables. Moderator variables (perceived racial/ethnic discrimination at school, school safety, neighborhood safety, and bullying at school) were a priori hypothesis driven by discussions with the students and the school district as factors affecting their overall health.

A general linear model (GLM) procedure was used to estimate the psychosocial stressors and sleep duration on mental health using hierarchical multiple regression analysis. The GLM procedure enables the degree of interaction and nested effects to be tested. In Step 1 of our regression model, we controlled for all covariates, whereas in Step 2, we entered our sleep variable. After adding the independent variable, sleep, the moderators (perceived racial/ethnic discrimination at school, school safety, neighborhood safety, and bullying at school) were added in Step 3. In Step 4, the model ran the interaction terms of the moderators and the sleep variables. These steps followed the methodology of hierarchical regression as ways to analyze variance in the outcome variable when the predictor variables are at varying hierarchical levels. This hierarchical linear regression was based on built sequential regression model where variables were added at each step to determine whether newly added variables show significant improvement in *R*‐squared (the proportion of explained variance in SMI by the model).

Approximately 20% of data was missing at random, and thus, to deal with this missingness, cases were deleted because given the size of the dataset, it was still robust.

### Ethical considerations

This study was approved by the Institutional Review Board of the organization of the authors. All students who participated provided parental informed consent and written informed assent. Privacy and anonymity of the participants were ensured by de‐coupling and de‐identifying the dataset used for the analysis.

## RESULTS

Of the eligible students, we had a response rate of 85% providing a total of 24,439 students participating in this study. As shown in Table 1, participants were predominantly Hispanic (78.5%), slightly over half were male (51%), and the majority of students (89.3%) reported being “free lunch” recipients revealing the socioeconomic disadvantage of the students. The average age for the students was 13.9 with the lowest age as 9 years and the oldest at 21 years. Grade level distributions, consisting of children and adolescents in 5th thru 12th grade, ranged from approximately 10%–14% with the lowest number of students being in grade 12 (*n* = 2576) and the highest in grade 6 (*n* = 3510).

**TABLE 1:** Demographic characteristics of California students in a low‐income serving school district in 2019–2020 (*n* = 24,439).

Characteristic	*n* (%)	*M*	SD	Range	Correlation with SMI: Pearson's (*r*) or Spearman's rho
K6: SMI		7.64	5.63	0–24	
Age		13.93	2.32	9.4–21.7	0.07***
Gender					
Male	12,516 (51.19)				0.11***
Female	11,932 (48.81)				
Race/ethnicity					
Black/African Americans	2548 (10.48)				
Hispanics	19,121 (78.53)				0.02***
Other races	1355 (5.57)				
White	1326 (5.45)				
Grade					
5th	3377 (13.83)				
6th	3510 (14.37)				
7th	3072 (12.58)				
8th	3079 (12.61)				0.06***
9th	3020 (12.36)				
10th	2933 (12.01)				
11th	2858 (11.70)				
12th	2576 (10.55)				
Free/reduced lunch					−0.007
No	2617 (10.70)				
Yes	21,831 (89.30)				
Sleeping with electronics					
No	6980 (29.17)				0.14***
Yes	16,951 (70.83)				
Hours of sleep		7.31	1.70	4–10	−0.26***
Perceived discrimination					
No	16,722 (86.74)				0.11***
Yes	2564 (13.26)				
Feel safe in their neighborhood					
No	2960 (16.34)				−0.19***
Yes	15,154 (83.66)				
Feel safe at school					
No	3827 (23.29)				−0.19***
Yes	12,603 (76.71)				
Bullied					
No	8586 (35.05)				0.28***
Yes	15,909 (64.95)				

Abbreviations: *M*, mean; SD, standard deviation; SMI, serious mental illness.

^***^ *p* < 0.001.

Overall, 18.7% of the students reported having SMI; however, distribution varied across grades. SMI increased with each grade level except in the 12 grade where it decreased slightly. Within each grade level, 13.6% of 5th graders, 13.7% of 6th graders, 17% of 7th graders, 18.7% of 8th graders, 20% of 9th graders, 22.5% of 10th graders, 24.5% of 11th graders, and 22.2% of 12th graders reported SMI. Gender, race/ethnicity, grade, sleeping with electronics, hours of sleep, perceived discrimination, being bullied and feeling safe in one's neighborhood and school were all correlated with SMI. Free/reduced lunch (indicator of socioeconomic status) was not correlated with SMI.

### Sleep

The average amount of sleep was 7 h with the range spanning from 4 to 10 h. Sleep deficit for school‐aged kids, ages 6–13 years (receiving less than the recommended 9–11 h) was highest among those who slept with electronics (69.6%) compared to those who did not (30.4%), (*p* < 0.001). Among adolescents (14–18 years), sleep deficit (less than the recommended 8–10 h) was highest among those who slept with electronics (76.9%) compared to those who did not (23.1%), *p* < 0.001. Seventy percent of the students slept with electronic devices such as an Xbox, a PlayStation, a smartphone, an iPad, or other tablet. Average sleep was 7.3 h with sleep deficit increasing with each grade level starting with 46.8% of 5th graders not getting enough sleep to 75.6% of 12th graders not getting enough sleep.

### Psychosocial stressors

With respect to perceived racial/ethnic discrimination at school, a small proportion (13%) of respondents indicated being disrespected or mistreated by an adult at their school because of their race, ethnicity, or nationality, 84% felt unsafe in their neighborhood where they lived, whereas just under one quarter felt unsafe at school, and nearly two thirds reported being bullied on school property. Of those who perceived racial/ethnic discrimination at school, 56.9% were males, 89.9% received free and reduced lunch; and of racial/ethnic background, 26.5% of Black/African American students, 11.4% of Hispanic student, 14.0% of other races, and 14.5% of White students perceived racial/ethnic discrimination at school.

Those with perceived racial/ethnic discrimination at school had more sleep deficit than those without (58.3% vs. 47.8%); those who felt unsafe in their neighborhood had more sleep deficit than those who did not (56.6% vs. 46.8%); those who felt unsafe at school had more sleep deficit than those who did not (60.5% vs. 48.5%). Among our sample population, 65% identified as being bullied at school. Among those bullied on school property, sleep deficit was higher in comparison to those not bullied (57.1% vs. 51.2%). All were statistically significant at *p* < 0.001.

### The relationship with psychosocial stressors

As shown in Table 2, our ordinary least squares regression analysis indicates that in Step 1 of our model demographic controls explained only 3% of the variance in SMI, with 11th graders and those who reported sleeping with electronics being significantly distressed and males and African Americans, having significantly less SMI. However, moving to Step 2, the addition of our sleep variable added an additional 6% variance to the model and affirms our first hypothesis that sleep duration would be inversely associated with SMI—hence as sleep increased SMI decreased. Of note, with the addition of sleep duration, in Step 2, the risk for SMI increased for fifth graders (from *β* = 0.02 to 0.19, *p* < 0.001).

**TABLE 2:** Hierarchical linear regression analysis examining relationship between California student sleep duration and serious mental illness (SMI) moderated by psychosocial factors (discrimination, safety, and bullying) in 2019–2020.

	Model 1		Model 2		Model 3		Model 4	
	*R* ^2^ = 0.03		*R* ^2^ = 0.09		*R* ^2^ = 0.17		*R* ^2^ = 0.18	
	*β*	CI	*β*	CI	*β*	CI	*β*	CI
Intercept	2.62***	2.17–3.07	3.94***	3.51–4.39	3.07***	2.46–3.68	2.96***	2.34–3.58
Gender								
Male	−0.22***	−0.3 to −0.2	−0.22***	−0.25 to −0.19	−0.17***	−0.21 to 0.13	−0.17***	−0.21 to −0.13
Female	1.00	Ref	1.00	Ref	1.00	Ref	1.00	Ref
Race/ethnicity								
African American	−0.18***	−0.25 to 0.10	−0.14***	−0.21 to −0.07	−0.05	−0.15 to 0.05	−0.05	−0.15 to 0.05
Hispanic	−0.06	−0.13 to 0.01	−0.03	−0.09 to 0.03	0.11*	0.03–0.20	0.11	0.03–0.19
Other races	−0.01	−0.09 to 0.07	0.01	−0.07 to 0.09	0.13*	0.01–0.24	0.13*	0.01–0.24
White	1.00	Ref	1.00	Ref	1.00	Ref	1.00	Ref
Grade								
5	0.02	−0.9 to 0.13	0.19***	0.07–0.29	0.11	−0.04 to 0.25	0.11	−0.04 to 0.26
6	−0.04	−0.12 to 0.04	0.11**	0.25–0.19	0.08	−0.03 to 0.19	0.08	−0.03 to 0.19
7	0.04	−0.10 to 0.03	0.03	−0.04 to 0.09	−0.00	−0.09 to 0.08	−0.00	−0.08 to 0.08
8	1.00	Ref	1.00	Ref	1.00	Ref	1.00	Ref
9	0.03	−0.03 to 0.93	−0.02	−0.08 to 0.04	−0.03	−0.12 to 0.05	−0.03	−0.12 to 0.06
10	0.06	−0.03 to 0.15	−0.03	−0.11 to 0.06	−0.04	−0.16 to 0.08	−0.04	−0.16 to 0.08
11	0.18**	0.06–0.29	0.05	−0.05 to 0.16	0.12	−0.04 to 0.27	0.12	−0.04 to 0.27
12	0.07	−0.07 to 0.21	−0.07	−0.22 to 0.06	0.04	−0.15 to 0.23	0.03	−0.16 to 0.23
Free and reduced lunch	−0.02	−0.06 to 0.03	−0.00	−0.05 to 0.04	0.01	−0.05 to 0.07	0.01	−0.05 to 0.07
Sleeping with electronics	0.05***	0.04–0.05	0.04***	0.03–0.04	0.02***	0.02–0.03	0.02***	0.02–0.03
Hours of sleep			−0.18***	−0.19 to −0.17	−0.16***	−0.17 to −0.14	−0.15***	−0.17 to 0.13
Discriminated against					0.13***	0.06–0.19	0.51***	0.26–0.75
Safe in neighborhood					0.32***	0.25–0.37	0.57***	0.34–0.81
Safe at school					0.23***	0.17–0.27	0.29***	0.07–0.50
Bullied					0.54***	0.49–0.58	0.46***	0.26–0.66
Sleep * discrimination							−0.05**	−0.08 to 0.02
Sleep * neighborhood safety							−0.04*	−0.06 to 0.00
Sleep * safety at school							−0.01	−0.03 to 0.02
Sleep * bullying							0.01	−0.02 to 0.04

Abbreviations: CI, confidence interval; *p*, *p*‐value; *R* ^2^, *R*‐squared; *β*, beta.

* *p* < 0.05, ^**^ *p* < 0.01, ^***^ *p* < 0.001.

With the addition of the psychosocial stressors in Step 3, there was an added 8% variance. An increased risk for SMI was significantly associated with perceived racial/ethnic discrimination at school *β* = 0.13, *p* < 0.001), feeling unsafe in one's neighborhood (*β* = 0.32, *p* < 0.001), feeling unsafe at school (*β* = 0.23, *p* < 0.001), and being bullied at school (*β* = 0.54, *p* < 0.001) even after controlling for sleep duration and demographics. With the addition of these psychosocial stressors, Hispanic and students of other races were found to be at an increased risk of SMI (*β* = 0.11, *β* = 0.13, *p* < 0.05) in comparison to Whites.

**FIGURE 1:**
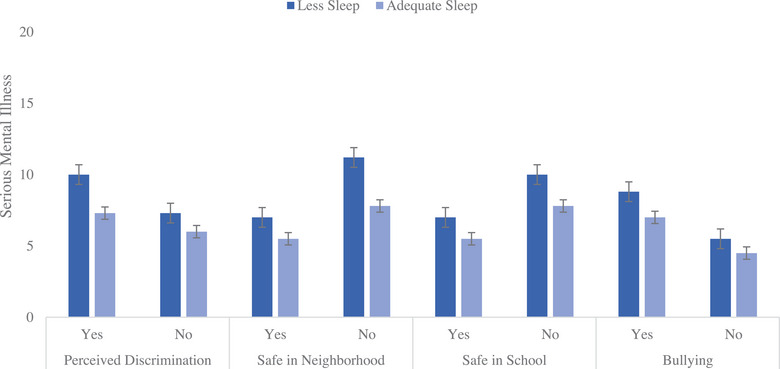
California children 13 years and younger in a low‐income serving school district (2019–2020) reportings of serious mental illness (SMI) by whether a student is getting adequate sleep or not when experiencing psychosocial stressors.

**FIGURE 2:**
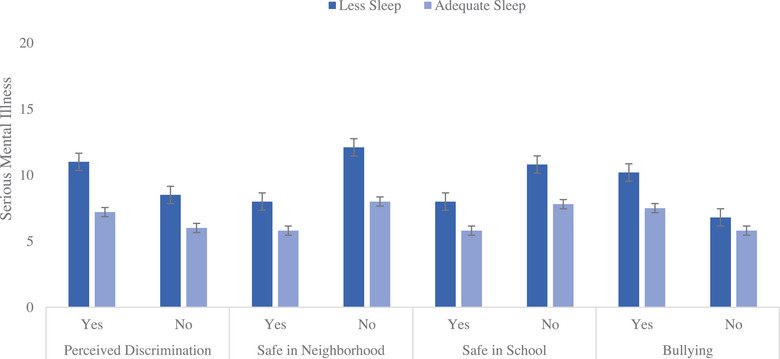
California children 14 years and older in a low‐income serving school district (2019–2020) reportings of serious mental illness (SMI) by whether a student is getting adequate sleep or not when experiencing psychosocial stressors.

The addition of interaction (Step 4) terms for sleep and the psychosocial stressors (perceived racial/ethnic discrimination at school, bullying at school, safety at school and in the neighborhood) added 1% additional variance to the model. Both perceptions of discrimination (*β* = −0.05, *p* < 0.01) and neighborhood safety (*β* = −0.04, *p* < 0.05) moderated the relationship between sleep duration and SMI so that the relationship between shorter sleep duration and increased SMI was worse for those perceiving discrimination and feeling unsafe in their neighborhoods. Interaction bar graphs (Figures 1 and 2) depict SMI by whether a student in getting adequate sleep or not when they report experiencing the various psychosocial stressors (perceived discrimination, safety in the neighborhood, safety in school, and bullying). Both figures reveal that those not experiencing psychosocial stressors have significantly less SMI and those receiving adequate sleep also have less SMI.

Overall, not only did the proposed psychosocial stressors (perceived racial/ethnic discrimination at school, bullying at school, safety at school, and in the neighborhood) increase the *R*‐squared (*R* ^2^ = 0.17, *p* < 0.001) but also so did the interactions with sleep in model 4 (*R* ^2^ = 0.18, *p* < 0.001).

## DISCUSSION

We found that SMI (primarily measured as internalizing symptoms) was disproportionately higher in our sample than studies of White children and adolescents, but in line with previous studies of underserved minoritized youth [47, 48]. Furthermore, our study reveals that SMI increases as students become older. In our sample SMI increased from 13.6% among 5th graders to 24.5% among 11th graders.

Although boys and young men have substantially worse mental health than girls and young women [49], our study revealed that among children and adolescents from racially diverse and low‐income backgrounds, the risk for SMI symptoms among boys is lower in comparison to the participating females. This difference could be attributed to the fact that females are more likely than their male counterparts to disclose depressive or mental health symptoms [50]. In addition, more girls and young women reporting SMI is also in alignment with a Centers for Disease Control and Prevention report that showed that three in five girls feel persistently sad and hopeless—a marker for depressive symptoms [51]. This study also identified that SMI is present among fifth graders and could potentially be present in younger grades as well. Children in this age group and younger are undergoing critical physical milestones (growth spurts, puberty, increased appetite, and need for sleep), cognitive milestones (thinking changes from what they can observe to abstract ideas, develop better sense of responsibility, and memory skills begin to increase), and social–emotional changes (insecure or have mood swings, test limits and push boundaries, finding their own talents, begin having a deeper understanding of relationships) [52, 53]; and when they live in low‐income, stressful home environments, the risk for emotional and behavioral problems increases [54]. With the onset of depression presenting itself at earlier ages and increasing with age [55], targeted interventions for this age group are called for.

Additionally, in agreement with literature, our study affirmed that the use of electronics in bed not only disrupts sleep but also affects one's risk for SMI [46]. This is problematic for children and adolescents for loss of even half an hour of sleep negatively impacts their behavior the next day [56, 57]. Issues appear to begin at younger ages and progressively get worse as students get older (grade being a proxy for age), indicating that interventions should begin at younger ages. In all grades, it was also found that sleeping with electronics was positively correlated with SMI, and less sleep was associated with SMI. Regardless of grade, bullying had the strongest effect to SMI; however, the effect varied with each grade level with the smallest effect at sixth grade and the largest effect at seventh grade.

The presence of the psychosocial stressors, specifically perceived discrimination and neighborhood safety, was found to moderate the effect of sleep on SMI so that the relationship between shorter sleep duration and increased SMI was worse. According to the American Academy of Sleep Medicine, teenagers between the ages of 13 and 18 years of age should sleep 8–10 h per 24 h on a regular basis to promote optimal health [58]. For this population, we observed an inverse linear relationship between sleep risks for SMI. Hence, increased sleep among the children and adolescents was associated with reduced SMI; however, in the presence of psychosocial stressors, the effect of sleep on SMI was moderated. Discrimination, bullying, and safety have been shown in independent studies to be linked to poor sleep [59, 60] and SMI [61, 62, 63]; however, this study shows the relationship of all these factors together. Ultimately, despite increased sleep, experiencing psychosocial stressors was found to increase one's SMI.

Despite our results, it is worth noting that discrimination, bullying at school, and safety do not explain all the SMI experienced by these students as seen by the low statistical variance presented in each model. The low variance could also be because factors, such as bullying and discrimination, were only measured in a school setting, other settings may also explain some additional variance. Studies have shown that exposures to adverse childhood experiences [64], social disadvantages [65], substance abuse [66], and long‐term stress [67] are correlated with increases with SMI. Additionally, with the increasing frequency of mass shootings [6] and the threat of detention or deportation among undocumented families [68] and COVID‐19, children and adolescents suffer an increased psychological toll increasing the incidence of a range of negative mental health outcomes. A deeper analysis on the student's multilayered ecology (i.e., psychological, biological, social, and cultural/contextual) is needed to understand fully the root of their SMI.

### Implications for school health policy, practice, and equity

This study provides insights for improving school health in the following ways: first by recognizing that early experiences play a vital role in shaping brain development and laying the foundations of sound mental health. Disruptions to this developmental process can and do occur in young children, whereas diagnosis in early childhood can be difficult, and it is essential that treatment happens specifically in the context of a child's/adolescent's family, home, and community. Second, it is important to ensuring that students have the opportunity to have adequate sleep. Schools can educate both students and parents on the importance of sleep. It is also important for schools to consider their start times to ensure that students are not starting school too early. Finally, it is essential to implement mental health interventions that address structural racism and discrimination affecting children and adolescents of low‐income and minoritized backgrounds is critical. These interventions should span various socioecological domains (school level, neighborhood/community, and societal) in order to improve minority health and promote health equity.

### Limitations

The results of this study should be interpreted in light of several limitations. First, we relied on self‐report measures. Additionally, due to the cross‐sectional nature of this study, variables were only measured at one point in time, not allowing for causal inferences to be made. Moreover, some of the measures used had different time frames, for instance, SMI (30 days), bullying (12 months), and sleep duration (on an average sleep night). These questions were pulled from various surveys for the purposes of comparing this district to California and National statistics, and therefore, questions could not be changed. However, most of the questions were asked in the context of a school time frame. Due to time limitations, measures for discrimination and sleep consisted of only one question as opposed to validated larger scales. Our questions, however, demonstrate good test–retest reliability [69]. It would be useful for future studies to replicate our findings using more comprehensive measures of sleep, mental health, and discrimination.

Nonetheless, in contrast to previous national studies typically having small samples of minority students [70], our sample possessed 10.4% Black, 78.5% Hispanics, and 5.45% of other races, providing an excellent opportunity to examine the dynamics of the racial gaps even when minorities are the majority. Finally, our measure of SMI primarily assessed for internalizing symptoms (e.g., nervous, hopeless, and depressed). Future research is needed to fully understand how these findings may be generalized to more externalizing symptoms.

## CONCLUSION

This study demonstrates that increased hours of sleep among children and adolescents were associated with reduced risk of SMI. However, in the presence of psychosocial stressors (discrimination, bullying, and perceived school and neighborhood safety), the effect of sleep on SMI was moderated, and despite increased sleep, experiencing psychosocial stressors was found to increase one's SMI. Targetted interventions are called for among children and adolescents to target their SMI and the psychosocial stressors that exacerbate SMI.

## AUTHOR CONTRIBUTIONS

*Conceptualization; supervision; writing—original draft; writing—review and editing*: Nipher Malika, Tori R. Van Dyk, and Susanne Montgomery. *Data curation; writing—original draft; writing—review and editing*: Qais Alemi. *Conceptualization; writing—review and editing*: Juan Carlos Belliard. *Writing—review and editing*: Catherine Fisher. *Conceptualization; writing—original draft*: Larry Ortiz.

## CONFLICT OF INTEREST STATEMENT

The authors declare that there is no conflict of interest that could be perceived as prejudicing the impartiality of the research reported.

## HUMAN SUBJECTS APPROVAL STATEMENT

This study was approved by Loma Linda University Institutional Review Board and all participants provided written informed assent and informed consent from parents/guardians.

## ETHICS STATEMENT

This study was approved by the author's‐affiliated Institutional Review Board, and all participants provided written informed assent and informed consent from parents.

## Data Availability

The data that supports the findings of this study is available upon email request from the corresponding author.
